# Identification of an m6A Regulators-Mediated Prognosis Signature For Survival Prediction and Its Relevance to Immune Infiltration in Melanoma

**DOI:** 10.3389/fcell.2021.718912

**Published:** 2021-11-25

**Authors:** Liuxing Wu, Xin Hu, Hongji Dai, Kexin Chen, Ben Liu

**Affiliations:** Department of Epidemiology and Biostatistics, Key Laboratory of Cancer Prevention and Therapy of Tianjin, Key Laboratory of Molecular Cancer Epidemiology, National Clinical Research Center for Cancer, Tianjin’s Clinical Research Center for Cancer, Tianjin Medical University Cancer Institute and Hospital, Tianjin, China

**Keywords:** signature for prognosis guidance, immune infiltration, melanoma, m6A clusters, expression pattern of m6A regulators

## Abstract

Despite robust evidence for the role of m6A in cancer development and progression, its association with immune infiltration and survival outcomes in melanoma remains obscure. Here, we aimed to develop an m6A-related risk signature to improve prognostic and immunotherapy responder prediction performance in the context of melanoma. We comprehensively analyzed the m6A cluster and immune infiltration phenotypes of public datasets. The TCGA (*n* = 457) and eleven independent melanoma cohorts (*n* = 758) were used as the training and validation datasets, respectively. We identified two m6A clusters (m6A-clusterA and m6A-clusterB) based on the expression pattern of m6A regulators *via* unsupervised consensus clustering. *IGF2BP1* (7.49%), *KIAA1429* (7.06%), and *YTHDC1* (4.28%) were the three most frequently mutated genes. There was a correlation between driver genes mutation statuses and the expression of m6A regulators. A significant difference in tumor-associated immune infiltration between two m6A clusters was detected. Compared with m6A-clusterA, the m6A-clusterB was characterized by a lower immune score and immune cell infiltration but higher mRNA expression-based stemness index (mRNAsi). An m6A-related risk signature consisting of 12 genes was determined via Cox regression analysis and divided the patients into low- and high-risk groups (*IL6ST, MBNL1, NXT2, EIF2A, CSGALNACT1, C11orf58, CD14, SPI1, NCCRP1, BOK, CD74, PAEP*). A nomogram was developed for the prediction of the survival rate. Compared with the high-risk group, the low-risk group was characterized by high expression of immune checkpoints and immunophenoscore (IPS), activation of immune-related pathways, and more enriched in immune cell infiltrations. The low-risk group had a favorable prognosis and contained the potential beneficiaries of the immune checkpoint blockade therapy and verified by the IMvigor210 cohort (*n* = 298). The m6A-related signature we have determined in melanoma highlights the relationships between m6A regulators and immune cell infiltration. The established risk signature was identified as a promising clinical biomarker of melanoma.

## Introduction

Melanoma is a highly malignant tumor characterized by invasive and expansive growth, a high relapse rate, a short survival time, and a high grade. Molecular pathological aberrations have been widely studied in melanoma and have led to the development of multiple therapeutic modalities, including immunotherapy. At present, drugs for melanoma include small molecule inhibitors for *BRAF* or *MEK*, anti-*CTLA4* antibody, anti-*PD1* antibody, and modified oncolytic herpes virus talimogene laherparepvec (T-VEC) (Luke et al., 2017). A study based on the national cancer registration data in England showed that cutaneous malignant melanoma incidence rate in females is higher than males in the young group, and a similar result was observed in the United States (Donley et al., 2019; Memon et al., 2021). Immune checkpoint blockade increases the survival rate for some patients (Allemani et al., 2018). Although immunotherapy has dramatically improved some patients’ prognosis, there remains a number of patients who have not responded to the drugs available for immunotherapy (Axelrod et al., 2018). Due to individual differences, it is more than necessary to identify biomarkers and apply individualized therapeutic approaches.

M6A is the most common modification of eukaryotic RNA (Dominissini et al., 2012). It plays a crucial role in RNA stability, mRNA precursor shearing, polyadenylation, mRNA transport and translation, noncoding RNAs cleavage, and degradation, which participate in the occurrence and development of many tumors (Arguello et al., 2017; Zhao et al., 2017; Wang et al., 2018; Yang et al., 2018). The m6A modification is reversible and regulated by three types of regulators, including methyltransferases, RNA binding proteins, and demethylases (Liu et al., 2020). The methyltransferases, also known as “writers,” including *METTL3/14*, *WTAP*, etc., catalyzes methyl transfer to the nitrogen atom in position six of adenylate. Then the inverse process is regulated by demethylases, also called “erasers,” including *FTO* and *ALKBH5*. The RNA binding proteins, including *YTHDF1-3*, *FMR1*, and so on, also referred to as “readers,” are recruited by the m6A site and specifically bind m6A residues (Liu J. et al., 2019). M6A has been shown to be associated with multiple cancers such as melanoma, gastric cancer, and hepatoblastoma (Liu L. et al., 2019; Yue et al., 2019). A recent study found that *METTL3*-mediated m6A modification promotes the proliferation and metastasis of uveal melanoma *via* the targeting of *c-Met* (Luo et al., 2020). *Hint2* has been considered as a tumor suppressor and promotes the Ca^2+^ pumping into the mitochondria. Such a process causes the apoptosis of mitochondria (Ndiaye et al., 2013). The m6A regulators inhibited melanoma development by increasing the methylation of *Hint2* (Jia et al., 2019). Yang et al. revealed the positive/negative regulatory role for *METTL3/ALKBH5* in *RHOB* and *RHOC* (a subset of small GTPase proteins) in melanoma (Yang et al., 2020). *YTHDF1* interferes with the presentation of dendritic cells by promoting the expression of lysosomal protease in dendritic cells and gives rise to disorder in the immune response. This work demonstrated that m6A modification constitutes an important regulator in immune infiltration (Han et al., 2019).

Mutations in driver genes can promote cancer development and progression, along with a significant impact, such as giving tumor cells selective advantages, improving cell division, avoiding apoptosis, and negative growth regulation (Martinez-Jimenez et al., 2020). However, the correlation between m6A regulators and driver genes remains unknown in melanoma.

Some studies have shown that tumor-initiating stem cells inhibit cytotoxic T cell responses *via* the surface molecule of *CD80*, which suggests that tumor-initiating stem cells could be essential to activating immune checkpoint pathways (Miao et al., 2019). M6A is involved in regulating many signaling pathways associated with stem cell differentiation (Lv et al., 2018; Su et al., 2020). The mRNA expression-based stemness index (mRNAsi) was used to estimate the degree of oncogenic dedifferentiation. The results showed that higher mRNAsi is correlated with a reduced immune infiltration, such as a lower leukocyte fraction and *PDL1* expression (Malta TM. et al., 2018). However, the association between m6A and mRNAsi is not clear in melanoma.

Immunotherapy has good therapeutic potential in the context of several fatal neoplasms, such as melanoma and non-small cell lung cancer (Schadendorf et al., 2012; Martinez et al., 2019). Yang et al. showed that *FTO* attenuates the clinical response to anti-*PD1* therapy in melanoma (Yang et al., 2019a). The study demonstrated that knockdown of *FTO* promotes the degradation of *PD1*, *CXCR4*, and *SOX10 via* increased methylation, and *YTHDF2* recognizes the process. The study of Li et al. revealed that small-molecule *ALKBH5* inhibitor increased the immune response in melanoma (Li et al., 2020). These studies suggested that combining target m6A regulator and anti-immune checkpoint therapy may improve the efficacy of immunotherapy in melanoma. However, the above studies were restricted to single m6A regulators. Little is known about the association between multiple m6A regulators and the immune infiltration, pathological characteristics, and clinical prognosis of melanoma. Our study identified a risk signature consisting of 12 m6A-related genes. In addition, we analyzed the association between m6A and infiltration in melanoma. The signature has been verified in four independent melanoma cohorts and an anti-*PDL1* cohort.

## Materials and Methods

### Data Acquisition

The gene expression data and clinicopathological data of 457 melanoma patients and 233 normal skin tissues were obtained from the UCSC Xena platform (http://xena.ucsc.edu/). Gene mutation maps of 457 melanoma patients were downloaded from TCGA (https://portal.gdc.cancer.gov/). Gene expression data and the clinical data of GSE65904 (*n* = 210), GSE59455 (*n* = 122), GSE54467 (*n* = 78), GSE91061 (*n* = 51), GSE19234 (*n* = 44), GSE78220 (*n* = 26), GSE22154 (*n* = 22) and GSE100797 (*n* = 21) were obtained from gene expression omnibus (GEO) (https://www.ncbi.nlm.nih.gov/geo/). Other gene expression data and the clinical data were obtained from previous studies, including a melanoma cohort from Nathanson et al. (*n* = 24), an anti-*CTLA4* melanoma cohort (*n* = 39), an anti-*PD1* melanoma cohort (n = 121), and IMvigor210 cohort (*n* = 298). Finally, 233 normal samples and 1,513 tumor samples were included in the follow-up analyses. The sequencing platform and sample size of each cohort are summarized in Table 1.

**TABLE 1:** The sequencing platform and sample size of each cohort.

Datasets	Citation	Platforms	Sample size
TCGA	Cancer Genome Atlas, (2015)	Illumina RNAseq HTSeq	457
GSE65904	Cirenajwis et al. (2015)	Illumina HumanHT-12 V4.0 expression beadchip	210
GSE59455	Budden et al. (2016)	Illumina HumanRef-8 WG-DASL v3.0	122
GSE54467	Jayawardana et al. (2015)	Illumina HumanWG-6 v3.0 expression beadchip	78
GSE91061	Riaz et al. (2017)	Illumina Genome Analyzer (Homo sapiens)	51
GSE19234	Bogunovic et al. (2009)	Affymetrix Human Genome U133 Plus 2.0 Array	44
GSE78220	Hugo et al. (2016)	Illumina HiSeq 2000 (Homo sapiens)	26
Melanoma cohort from Nathanson et al	Nathanson et al. (2017)	Illumina TruSeq mRNA library kit	24
GSE22154	Jönsson et al. (2010)	Illumina HumanHT-12 V3.0 expression beadchip	22
GSE100797	Lauss et al. (2017)	Illumina HiSeq 2000 (Homo sapiens)	21
Anti-*PD1*-melanoma cohort	Liu et al. (2019c)	Illumina’s TruSeq RNA Access Library Prep kit	121
Anti-*CTLA4*-melanoma cohort	Van Allen et al. (2015)	NA	39
IMvigor210	Mariathasan et al. (2018b)	NA	298

### Selection of m6A Regulators

A total of 21 regulators related to m6A modification were included in the follow-up analyses. These 21 m6A regulators included eight writers (*METTL3*, *RBM15*, *METTL14*, *RBM15B*, *CBLL1*, *WTAP*, *KIAA1429*, *ZC3H13*), eleven readers (*YTHDF1*, *YTHDF2*, *YTHDF3*, *YTHDC1*, *YTHDC2*, *IGF2BP1*, *FMR1*, *HNRNPA2B1*, *ELAVL1*, *HNRNPC*, *LRPPRC*) and two erasers (*ALKBH5*, *FTO*) (Zhang and Wu, 2020a).

### Differential Expression and Genetic Variation Analyses

The limma package was used to compare the difference in gene expression between normal samples and tumor samples. The mutation maps of the TCGA tumor samples were obtained, and the mutation waterfall map of the 19 mutated m6A regulators was drawn with the GenVisR package. The landscape of the CNV variation in the chromosome of these m6A regulators was plotted *via* R package OmicCircos. We acquired seven cutaneous melanoma driver genes from the previous study, included *BRAF*, *NRAS*, *CDKN2A*, *NF1*, *MECOM*, *COL5A1*, *DACH1*, in which mutation frequencies were more than 10% (Bailey et al., 2018). Then we performed a permutation test to examine the *p*-value of the expression of m6A regulators between the driver genes-mutant and -wild samples.

### Unsupervised Clustering of m6A Regulators and Gene Set Variation Analysis (GSVA)

Unsupervised clustering analysis of melanoma patients was performed using the ConsensusClusterPlus package in the R software. We used the GSVA enrichment analysis to explore the differences in pathways between the two m6A clusters. We downloaded the gene sets of “c2.cp.kegg.v7.4” from the MSigDB database for the GSVA enrichment analysis (Subramanian et al., 2005). FDR < 0.05 was considered statistically significant. We combined eight independent melanoma cohorts to validate the unsupervised clustering model, including GSE78220, GSE91061, GSE100797, a melanoma cohort from Nathanson et al. study, GSE65904, GSE19234, GSE22154, GSE59455.

### Evaluation of Tumor Immune Infiltration

We used the estimate R package to estimate the immune infiltration in TCGA melanoma patients. The 28 immune-related gene sets were obtained from the study of Charoentong et al. They contained both innate immune cells, such as eosinophils, mast cells, macrophages, and so on, and adaptive immune cells, such as CD4^+^ T cell, Gamma delta T cell, and so on (Charoentong et al., 2017a). The single-sample gene set enrichment analysis (ssGSEA) of 28 immune-related gene sets was used to quantify the abundances of 28 immune cells using GSVA package in melanoma patients. The estimate package was used to evaluate the immune scores, stromal scores, and tumor purity of the tumor patients. In addition, we utilized CIBERSORT, MCPcounter, TIMER algorithms to assess the abundances of immune infiltration between the two m6A-clusters. These three analyses were performed *via* the CIBERSORT function, MCPcounter, and IOBR R packages, respectively. Differential gene expression analysis between the two m6A clusters was performed *via* the limma package of the R language.

### Construction of an m6A-Related Signature and a Nomogram

We selected differential expression genes between two m6A clusters (*n* = 849, *p* < 0.05 and | log fold change | >0.5). We performed GO enrichment analysis for the differential expression genes *via* the clusterProfiler package. Then we screened genes present in both the GEO and our total of differentially expressed genes *via* univariate Cox regression analyses (*n* = 31, *p* < 0.0001). We used multivariate Cox regression analyses to narrow the range of target genes. The Akaike information criterion (AIC) was used to measure the prognostic prediction ability of the Cox proportional hazard regression model by calculating for different variables. Here, lower values of AIC indicate a better model fit. Finally, 12 genes were identified to establish a risk signature (Gaulke et al., 2019). The Benjamini and Hochberg method was applied for multiple-testing adjustments, and the *p*-value remained significant. The patients were divided into the high-risk and low-risk groups using the surv_cutpoint function (Chu et al., 2020). The integrated discrimination improvement (IDI) and the net reclassification improvement (NRI) were used to evaluate the performance of the signature (survIDINRI package). To test the predictive efficiency of this signature on immunotherapy, we performed the validation analysis through integrating five melanoma immunotherapy cohorts, including an anti-*PD1* melanoma cohort, an anti-*CTLA4* melanoma cohort, GSE91061, GSE78220 and GSE100797 (ICB-therapy-combined melanoma, *n* = 258). Data were corrected for batch effects *via* the combat function from the sva R package. To predict the survival rates, we constructed a nomogram model based on risk score, gender, stage, and age in 457 TCGA melanoma patients. We obtained the weights of the immune-related genes (four categories) from the previous study, including major histocompatibility complex (MHC)-related molecules, suppressor cells, effector cells, and checkpoints or immunomodulators. The weighted averaged Z-score was calculated based on the expression. Based on the sum of the weighted average Z-scores of four categories, the IPS was calculated (ranging from 0 to 10) (Charoentong et al., 2017b).

### Statistical Analysis

R 3.6.2 was used for data analysis and graph drawing. The detailed methods, software, algorithms, and sources of each cohort are summarized in Table 2. The expression correlations between 21 m6A regulators were analyzed by Spearman correlation coefficient analysis. Univariate Cox analysis was used to screen for genes that were significantly associated with prognosis. Then, a risk signature was constructed *via* the multivariable Cox regression model based on the stepwise regression with the minimum Akaike information criteria (AIC). A Wilcoxon rank-sum test was used to analyze the differences in continuous variables between the two groups. The log-rank test was used to compare the difference in survival rates between the two groups. Differences were established by chi-square tests and Fisher’s exact test. *p* < 0.05 was considered to be statistically significant.

**TABLE 2:** The detailed methods, software, algorithms and sources of each cohort.

Reagent or Resource	Source	Identifier
Software and algorithms		
Workflow	This paper	Figure 1
R 3.6.2	R Core Team (2019)	https://www.r-project.org/
limma (v 3.42.2)	Ritchie et al. (2015)	http://bioconductor.org/packages/release/bioc/html/limma.html
ConsensusClusterPlus (v 1.50.0)	Wilkerson and Hayes, (2010)	http://bioconductor.org/packages/release/bioc/html/ConsensusClusterPlus.html
GenVisR (v 1.18.1)	Skidmore et al. (2016)	http://bioconductor.org/packages/release/bioc/html/GenVisR.html
OmicCircos (v 1.24.0)	Hu et al. (2014)	http://www.bioconductor.org/packages/devel/bioc/html/OmicCircos.html
survminer (v 0.4.8)	Kassambara et al. (2020)	https://CRAN.R-project.org/package=survminer
GSVA (v 1.34.0)	Hänzelmann et al. (2013)	http://www.bioconductor.org/help/search/index.html?q=GSVA/
estimate (v1.0.13)	Yoshihara et al. (2013)	https://R-Forge.R-project.org/projects/estimate/
MCPcounter (v 1.2.0)	Becht et al. (2016)	https://github.com/ebecht/MCPcounter
IOBR (v 0.99.9)	Zeng et al. (2021)	https://github.com/IOBR/IOBR
CIBERSORT	Gentles et al. (2015)	https://precog.stanford.edu/
clusterProfiler (3.14.3)	Yu et al. (2012)	https://guangchuangyu.github.io/software/clusterProfiler/
ggplot2 (v 3.3.2)	Wickham, (2009)	https://cran.r-project.org/web/packages/ggplot2/index.html
sva (v 3.34.0)	Leek et al. (2012)	http://www.bioconductor.org/packages/release/bioc/html/sva.html
survIDINRI (v 1.1-1)	Uno et al. (2013)	https://CRAN.R-project.org/package=survIDINRI
Deposited Data		
Melanoma expression and clinical data	UCSC Xnea	https://xenabrowser.net/datapages/?hub=https://gdc.xenahubs.net:443
Melanoma somatic mutation data	The cancer genome atlas	https://portal.gdc.cancer.gov/

## Results

### The Landscape of Expression and Genetic Variation of m6A Regulators in Melanoma Patients

**FIGURE 1:**
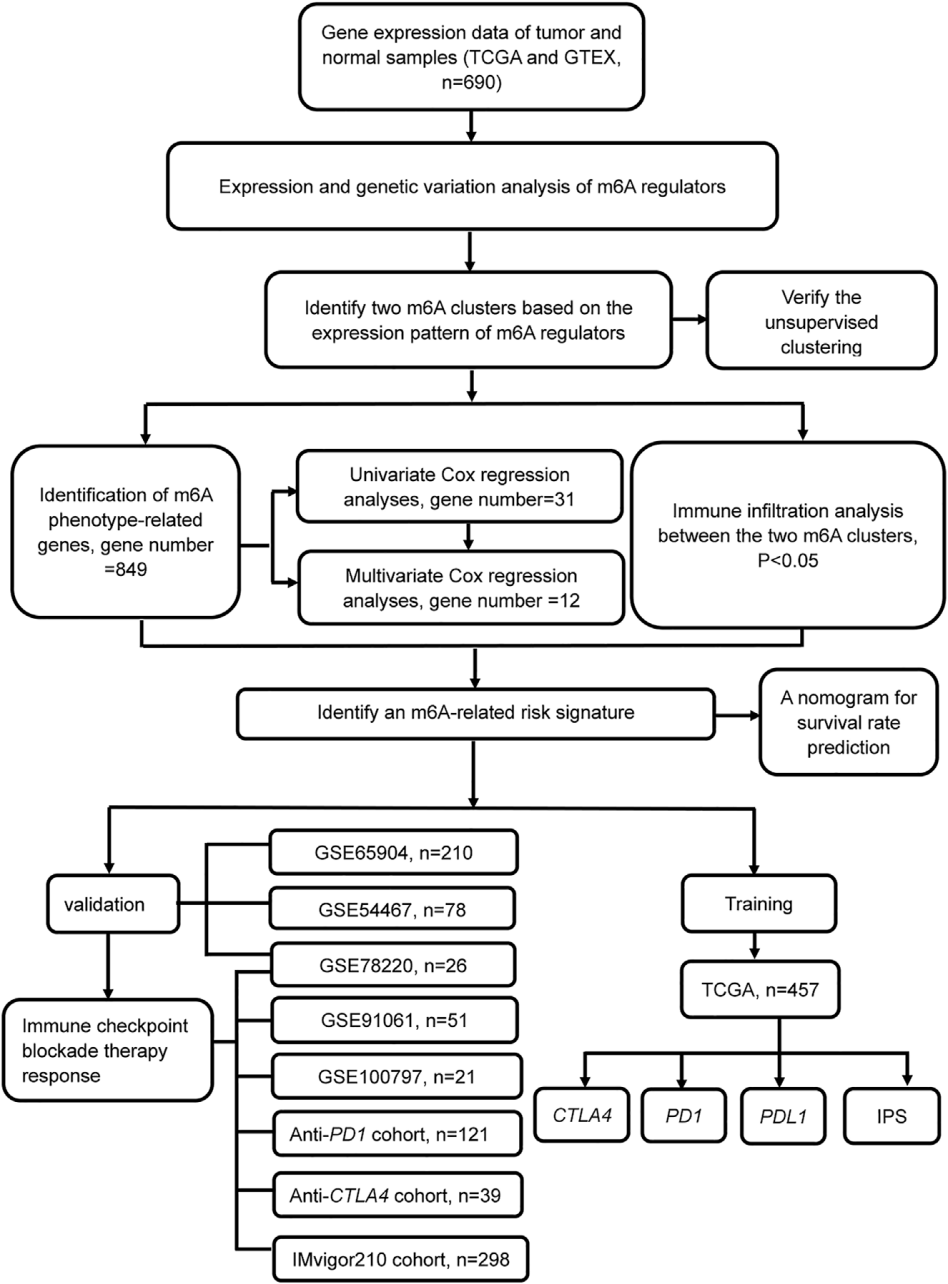
Flowchart of the article. Patients with survival information were selected for this study.

**FIGURE 2:**
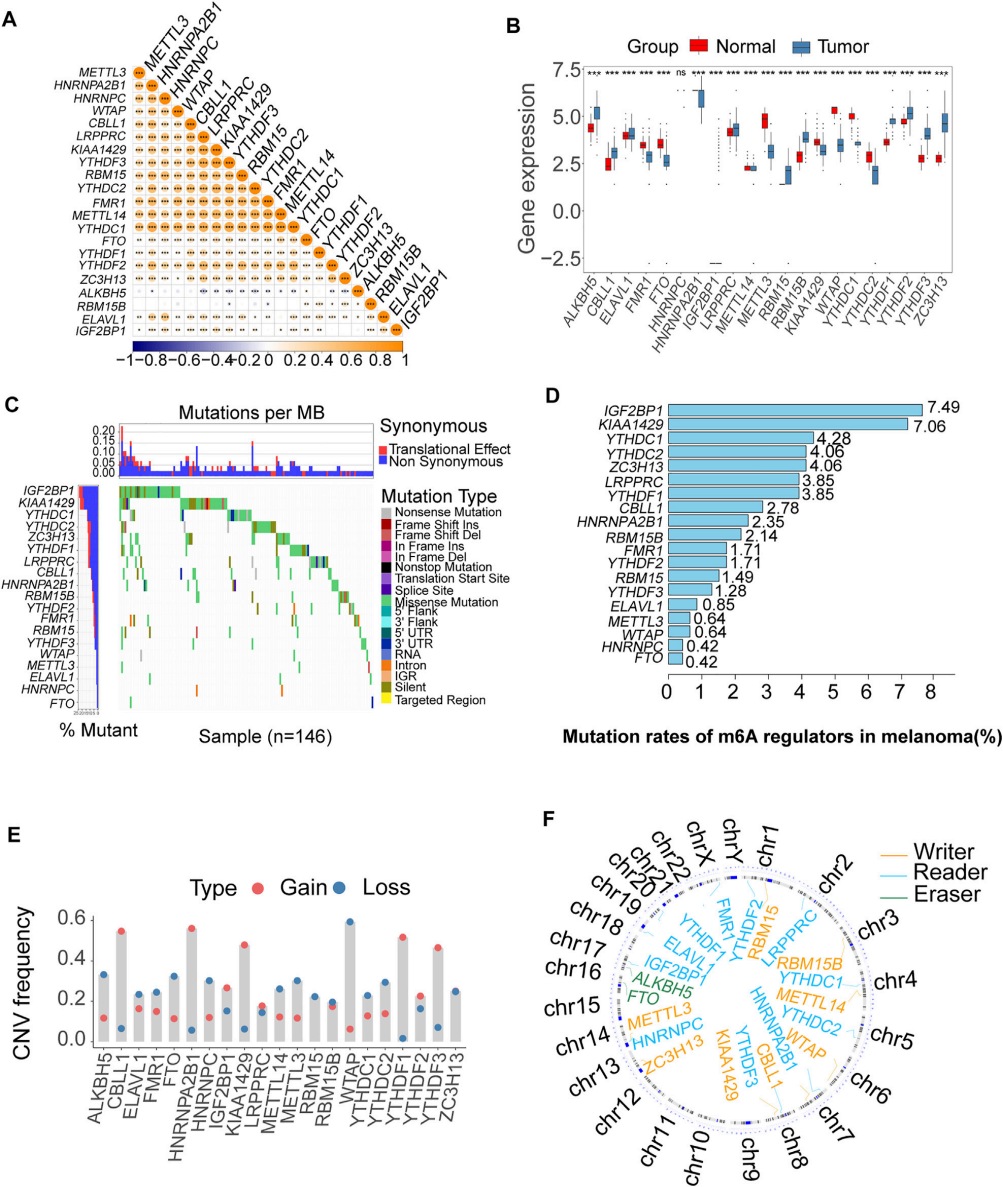
Bioinformatics analysis of the expression and genetic variation of m6A regulators in melanoma. **(A)** The expression correlation of m6A regulators; blue represents negative correlation, and orange represents positive correlation (*p < 0.05, **p < 0.01, ***p < 0.001). **(B)** The box plots were used to visualize the differential expression of 21 m6A regulators in tumor and normal samples; the expression values are log-transformed (*p < 0.05, **p < 0.01, ***p < 0.001). **(C)** Waterfall diagram of the m6A regulators in the TCGA melanoma cohort. Each column represented individual patients. The upper barplot showed TumorMutationBurden (TMB). The number on the left indicated the mutation frequency in each gene. **(D)** This bar graph displays the mutation rates of m6A regulators in TCGA melanoma. **(E)** The CNV alteration of the 21 m6A regulators. **(F)** The position of the CNV variation in the chromosome of these m6A regulators.

A schematic of the process is presented in Figure 1. The landscape of the expression correlation of these m6A regulators was shown in Figure 2A. We found most m6A regulators presented a significant positive correlation in expression, except for *ALKBH5*. To better understand the role of m6A regulators in the pathogenesis and progression of melanoma, we analyzed the differences in the expression of 21 m6A regulators between normal and tumor samples *via* the R package limma. Among the 21 m6A regulators, 11 were significantly more highly expressed in the tumor tissues than in the normal tissues (*RBM15B*, *RBM15*, *ALKBH5*, *YTHDF1-3*, *IGF2BP1*, *CBLL1*, *ELAVL1*, *LRPPRC*, and *ZC3H13*; *p* < 0.05, Figure 2B). Then, we analyzed the somatic mutations of 21 m6A regulators in TCGA melanoma; 19 m6A regulators displayed mutations, with the exception of only two genes (*METTL14* and *ALKBH5*) in melanoma tissues (Figure 2C). Among the 19 m6A regulators showing somatic mutations, *IGF2BP1*, *KIAA1429* and *YTHDC1* displayed the highest mutation frequency (7.49, 7.06, and 4.28%, respectively) (Figure 2D). To evaluate the potential signaling pathways controlling the mutation patterns of these three genes, we performed GO enrichment analysis in 67 melanoma cell lines using the mutation and RNA-seq expression profiles from the Cancer Cell Line Encyclopedia (CCLE) dataset (Ghandi et al., 2019). We found that the *IGF2BP1* and *KIAA1429* mutant cell lines exhibited a significant association with the functions related to DNA repair like melanin biosynthetic process and DNA strand elongation involved in DNA replication, as well as DNA replication and Mismatch repair. While Melanin metabolism pathway, including melanin biosynthetic process, melanosome organization, and Melanogenesis, were remarkably rich in the *YTHDC1* mutant cell lines (Supplementary Figure S1).

**FIGURE 3:**
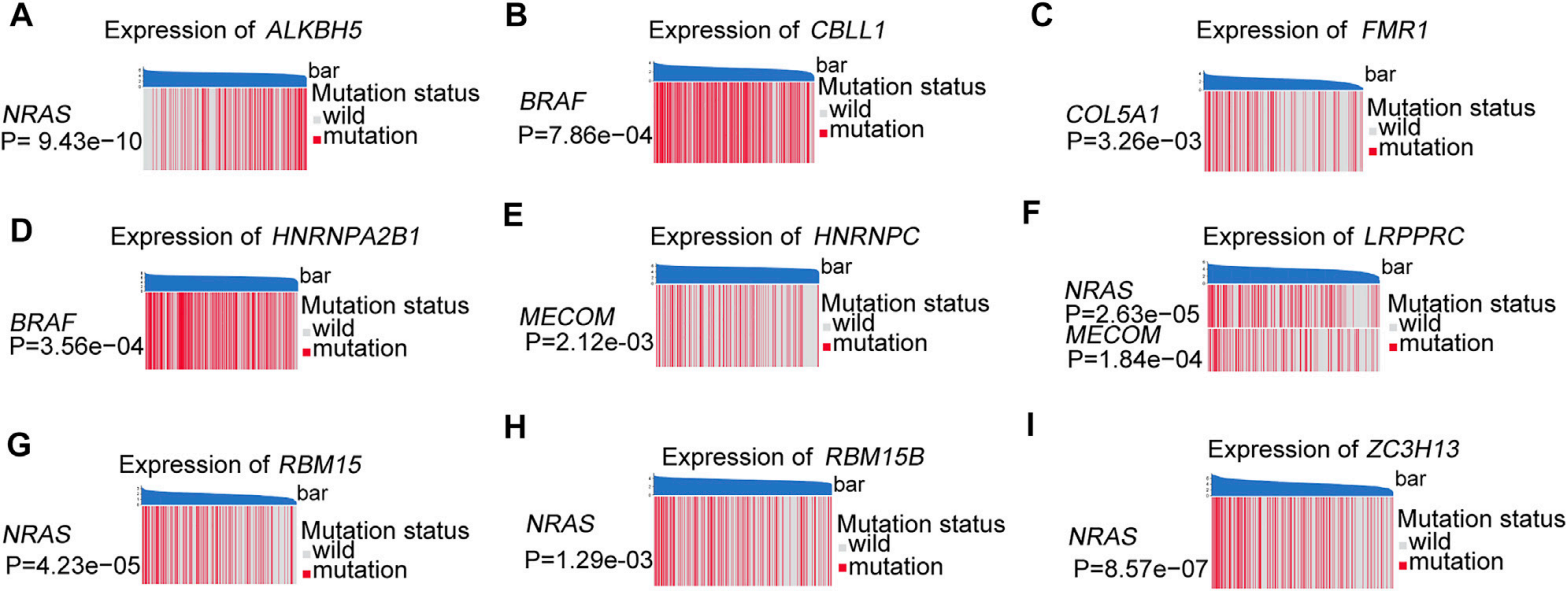
These heatmaps were used to visualize the expression of m6A regulators between the driver genes mutant and wild samples, permutation test, *p* < 0.005. **(A)** *ALKBH5* between the different mutation statuses of *NRAS*. **(B)** *CBLL1* between the different mutation statuses of *BRAF*. **(C)** *FMR1* between the different mutation statuses of *COL5A1*. **(D)** *HNRNPA2B1* between the different mutation statuses of *BRAF*. **(E)** *HNRNPC* between the different mutation statuses of *MECOM*. **(F)** *LRPPRC* between the different mutation statuses of *NRAS* and *MECOM*. **(G)** *RBM15* between the different mutation statuses of *NRAS*. **(H)** *RBM15B* between the different mutation statuses of *NRAS*. **(I)** *ZC3H13* between the different mutation statuses of *NRAS*.

The somatic copy number alteration (CNV) analysis revealed that most m6A regulators displayed deletion in copy number, except for *CBLL1*, *HNRNPA2B1*, *IGF2BP1*, *KIAA1429*, *LRPPRC*, and *YTHDF1-3* (Figure 2E). The position of the CNV variation in the chromosome of these m6A regulators is shown in Figure 2F. The high expression of eight m6A regulators (*CBLL1*, *FMR1*, *HNRNPA2B1*, *HNRNPC*, *LRPPRC*, *RBM15*, *RBM15B*, *ZC3H13*) was enriched in driver genes-mutated samples. The low expression of one m6A regulator (*ALKBH5*) was enriched in driver genes-mutated samples (permutation test, *p* < 0.005, Figure 3). These results showed the high heterogeneities of m6A regulators and indicated that the dysregulation of m6A regulators had a critical role in the pathogenesis and progression of melanoma.

### Identification of Two m6A Clusters Based on the Expression Pattern of m6A Regulators

**FIGURE 4:**
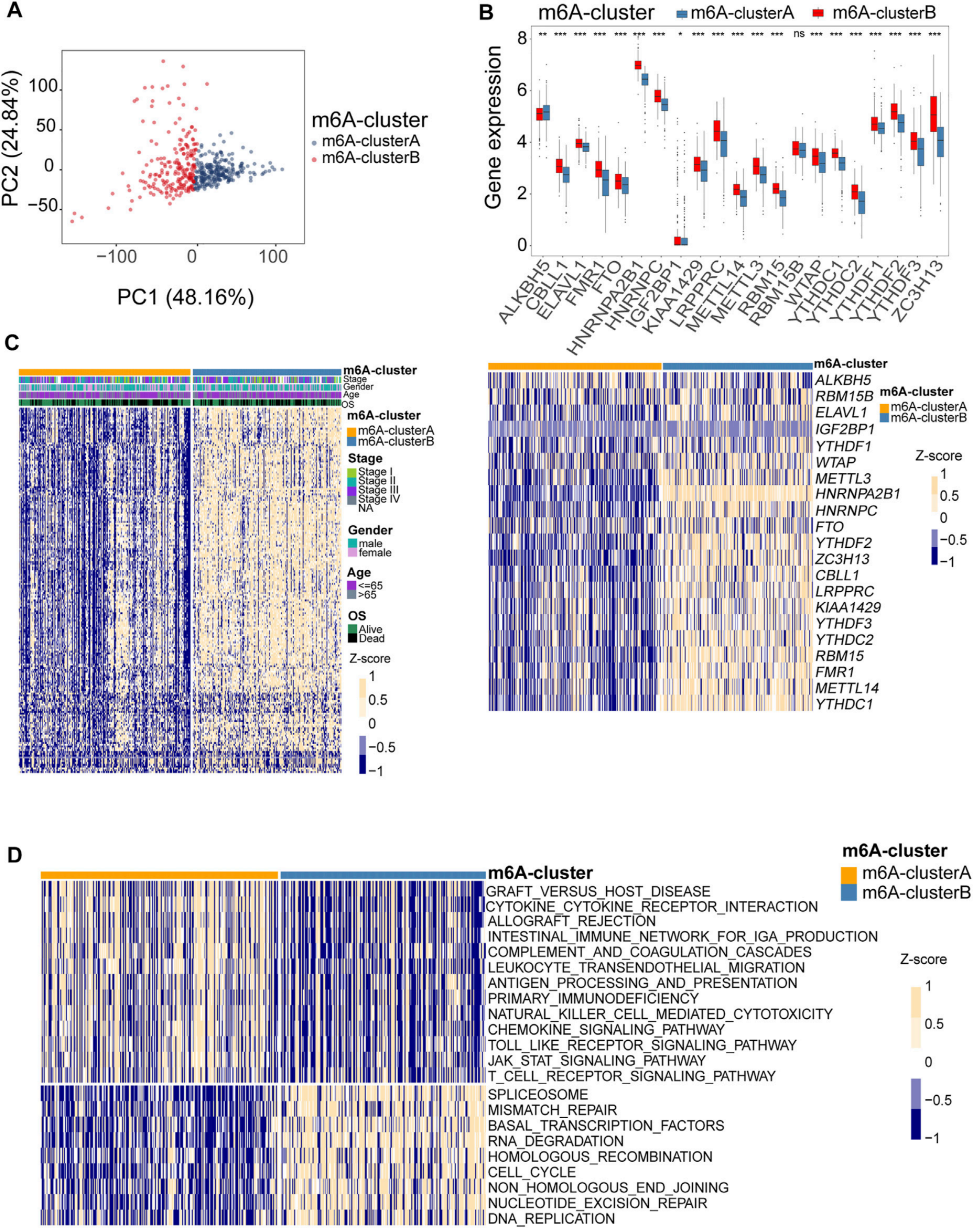
Identification of consensus clusters by 21 m6A regulators. **(A)** Principal component analysis indicated that there was a significant difference in transcriptome between the two m6A clusters. **(B)** Box plots were used to visualize the differential expression of 21 m6A regulators in two clusters (upper, *p < 0.05, **p < 0.01, ***p < 0.001). The heatmap shows the Z-score-transformed expression levels of 21 m6A regulators between the two m6A clusters **(down)**. **(C)** clinicopathological manifestations between the two m6A-clusters. **(D)** The heatmap shows the activation states of pathways between the two m6A clusters, and yellow represents activated and blue represents repressive pathways.

To comprehensively analyze the role of multiple m6A regulators in melanoma, we performed unsupervised clustering of the tumor samples using the expression values of 21 m6A regulators *via* the R package ConsensusClusterPlus. The consensus clustering algorithm was used to determine the most stable consensus clusters. According to the consensus matrix, when k = 2, the number of patients was evenly distributed in each cluster, without any cluster containing an exceptionally high or low number of patients. The clusters showed a low correlation. When K = 2, the CDF curve is flat, revealing the correct K (Wilkerson and Hayes, 2010; Senbabaoglu et al., 2014; Hu et al., 2021). The maximum Calinski-Harabasz (CH) index indicated that K = 2 was the optimal number of clusters (Supplementary Figure S2). Principal component analysis indicated a significant difference in transcriptome between the two clusters (Figure 4A). Two optimum clusters of m6A were obtained, named m6A-clusterA and m6A-clusterB, respectively (Figure 4B). Heatmap demonstrates the clinicopathological manifestations between the two m6A-clusters (Figure 4C). The association between the m6A clusters and the clinical indexes was analyzed, and we found that the low expression of *FMR1*, *METTL3* and *YTHDC2* was associated with the m6A-clusterA (Supplementary Table S1). To further understand the differences in biological processes between the two clusters, we employed GSVA enrichment analysis. The results indicated that the m6A-clusterA exhibited a significant association with the pathways related to immunological responses, such as antigen processing and presentation, natural killer cell-mediated cytotoxicity, chemokine signaling pathway, complement and coagulation cascades, and so on (Figure 4D). To verify the findings in the TCGA melanoma dataset, we performed external validation using a combined melanoma cohort to validate the unsupervised clustering model (*n* = 520). The result is consistent with the model based on TCGA, and K = 2 was the optimal value (Supplementary Figure S3).

### Different Characteristics of Immune Infiltration in Two m6A Clusters

**FIGURE 5:**
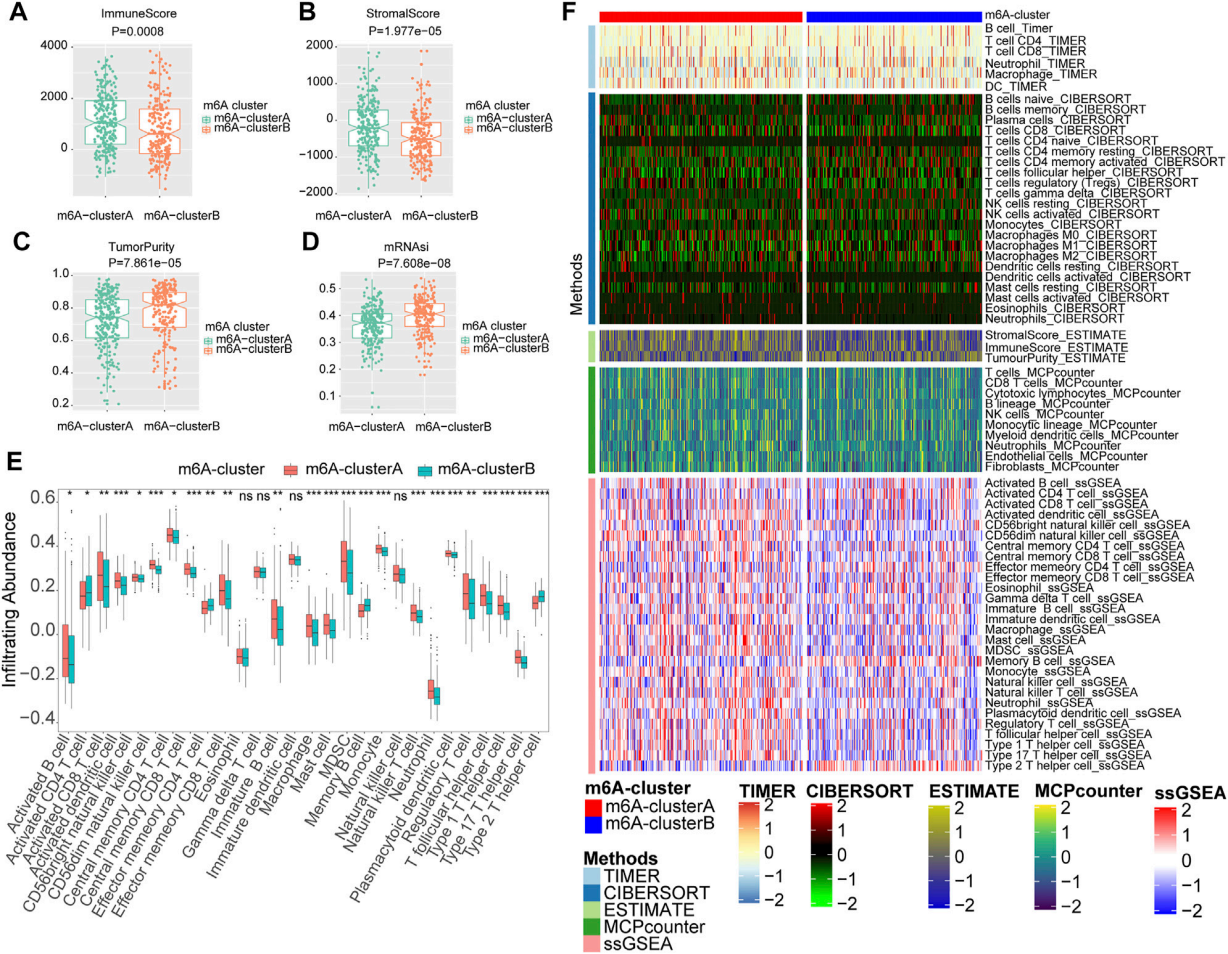
The two m6A clusters show differential immune infiltration. **(A)** Immune scores in melanoma samples were compared between the two m6A clusters. **(B)** Stromal scores in melanoma samples were compared between the two m6A clusters. **(C)**Tumor purity in melanoma samples was compared between the two m6A clusters. **(D)** MRNAsi in melanoma samples were compared between the two m6A clusters. **(E)** Infiltrating abundances of 28 immune cells were compared between the two m6A clusters (Wilcoxon test, *p < 0.05, **p < 0.01, ***p < 0.001) **(F)** The landscape of immune infiltration based on TIMER, CIBERSORT, estimate, MCP counter, and ssGSEA algorithms.

The correlation between the m6A RNA methylation regulators and immune infiltration was further evaluated. To assess the different immunological responses between the two m6A clusters, we measured the immune score, tumor purity, stromal score, mRNAsi, and infiltrating abundances of 28 immune cells using the Wilcoxon rank-sum test. The mRNA expression-based stemness index (mRNAsi) was obtained from the study of Malta et al. It was calculated with an innovative one-class logistic regression machine-learning algorithm (OCLR) based on mRNA expression, giving values between 0 and 1 (Malta TM. et al., 2018). The results showed that the m6A-clusterA had a higher immune score (Figure 5A) and stromal score (Figure 5B) but lower tumor purity (Figure 5C) and mRNAsi (Figure 5D) compared with the m6A-clusterB (Wilcoxon rank-sum test, *p* < 0.05). The infiltrating abundances of 28 immune cells indicated that most immune cells were enriched in the m6A-clusterA compared with the m6A-clusterB except for the Activated *CD4* T cell, Effector memory *CD4* T cell, Memory B cell, Type 2 T helper cell, Eosinophil, Gamma delta T cell, Immature dendritic cell, Natural killer cell (Wilcoxon test, *p* < 0.05, Figure 5E). Similar to previous conclusions, the m6A-clusterA was characterized by a higher immune score and immune cell infiltration in the validation cohort (Supplementary Figure S4).

In addition, we also utilized CIBERSORT, MCPcounter, and TIMER algorithms to evaluate the abundances of tumor immune infiltration. The heatmap was used to show the landscape of immune infiltration based on these five algorithms (Figure 5F). These results showed that multiple m6A regulators-mediated modification patterns might involve regulating tumor immune infiltration of melanoma.

### Identification of m6A-Related Risk Signature With Prognostic Significance and a Nomogram Construction

To predict the prognosis and guide individualized treatment, we used the differentially expressed genes with prognostic significance between the two m6A clusters to construct an m6A-related signature. We selected differential expression genes between two m6A clusters (*n* = 849, *p* < 0.05 and | log fold change | >0.5) (Supplementary Table S2). Then we performed GO enrichment analysis for these differential expression genes. These differential expression genes were enriched in m6A-related pathways, such as RNA splicing, regulation of mRNA processing, and so on (Supplementary Figure S5). So we considered these differential expression genes as m6A phenotype-related genes, which can be used to construct the m6A-related signature (Zhang et al., 2020b). Then we screened differential genes with prognosis significance *via* univariate Cox regression analyses (*n* = 31, *p* < 0.0001) (Supplementary Table S3). Moreover, 12 genes were used to construct the optimized risk signature with minimum Akaike information criterion (AIC) value *via* multivariate Cox regression analysis (*IL6ST, MBNL1, NXT2, EIF2A, CSGALNACT1, C11orf58, CD14, SPI1, NCCRP1, BOK, CD74, PAEP*) (Sun et al., 2020). The risk scores were derived from the following equations: Risk scores = ∑ (coefi × Xi), wherein coefi is the coefficient and xi is the relative expression value, transformed by Z-score, of the 12 genes (Supplementary Table S4).

**FIGURE 6:**
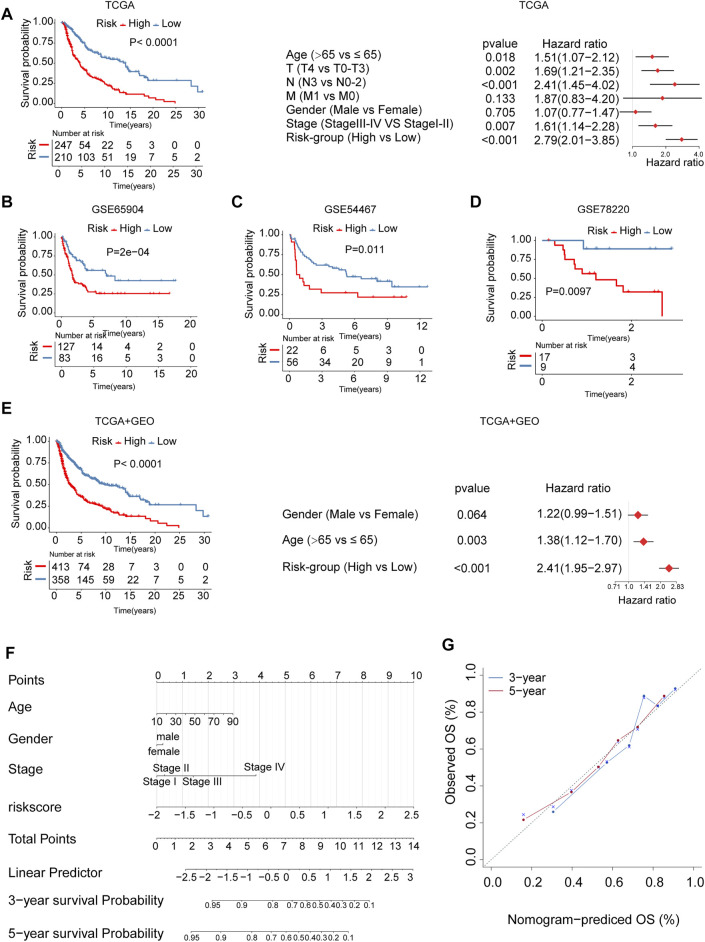
The m6A-related signature and the nomogram in the role of survival prediction. The Kaplan Meier plots for survival prediction of high-risk and low-risk. Forest plot representation of the multivariate Cox regression analysis of risk signature with age, gender, tumor stage was taken into account. **(A)** TCGA, **(B)** GSE65904, **(C)** GSE54467, **(D)** GSE78220, **(E)** combined TCGA-GEO. T: T-stage, primary tumor stage; N: N-stage, lymph node; M: M-stage, metastasis. **(F)** A Nomogram model was established using risk score and well-known risk factors. **(G)** The calibration curves showed favorable consistencies between the predicted and the actual survival probabilities in 3-, 5-years.

The patients in the cohort were divided into high- and low-risk groups using the surv_cutpoint function. The Kaplan Meier survival curves showed significant differences in overall survival between the two risk groups. The above results were independently validated in three melanoma cohorts from the gene expression omnibus database (GEO) (log-rank test, *p* < 0.05, Figures 6A–D). We plotted receiver operating characteristic curves (ROC curves) using timeROC in the R package. We evaluated the prediction accuracy of the risk signature according to the area under these ROC curves (AUC); high AUC values indicate good sensitivity and specificity (Supplementary Figure S6). The association between risk signature and survival remained statistically significant after age, gender, and stage were taken into account [TCGA, HR: 2.79 (95% CI: 2.01–3.85), *p* < 0.001, Figure 6A; GSE65904, HR: 2.20 (95% CI: 1.43–3.36), *p* < 0.001, Supplementary Figure S7A; GSE54467, HR:2.70 (95% CI: 1.41–5.20), *p* = 0.003, Supplementary Figure S7B; GSE78220, HR: 10.49 (95% CI: 1.30–84.15), *p* = 0.027, Supplementary Figure S7C; TCGA + GEO: HR: 2.41 (95% CI: 1.95–2.97), *p* < 0.001, Figure 6E]. We calculated the integrated discrimination improvement (IDI) and the net reclassification improvement (NRI) for the comparation of the old model (AJCC stage only, without risk score) and combined model (combined stage and risk score). The IDI for 1- and 3-years were 5.5% (*p* < 0.001) and 16.5% (*p* < 0.001), respectively. Consistently, the NRI for 1- and 3-years were 30.2% (*p* = 0.006), 40.1% (*p* < 0.001), respectively, (Supplementary Figures S8A,B). From the ROC curves, the AUCs of combined model for the survival prediction in 1- and 3-years were 0.721 and 0.745, respectively, higher than the old model (Supplementary Figures S8C,D). In addition, we developed a nomogram based on risk score, age, gender, and stage for survival rate prediction (Figure 6F). The C-index of the nomogram was 0.726 (95% CI, 0.685–0.767), markedly higher than AJCC stage (0.621, 95% CI: 0.580–0.662). A calibration curve at 3 or 5 years showed high consistency between predicted survival probability and actual survival proportion (Figure 6G).

**FIGURE 7:**
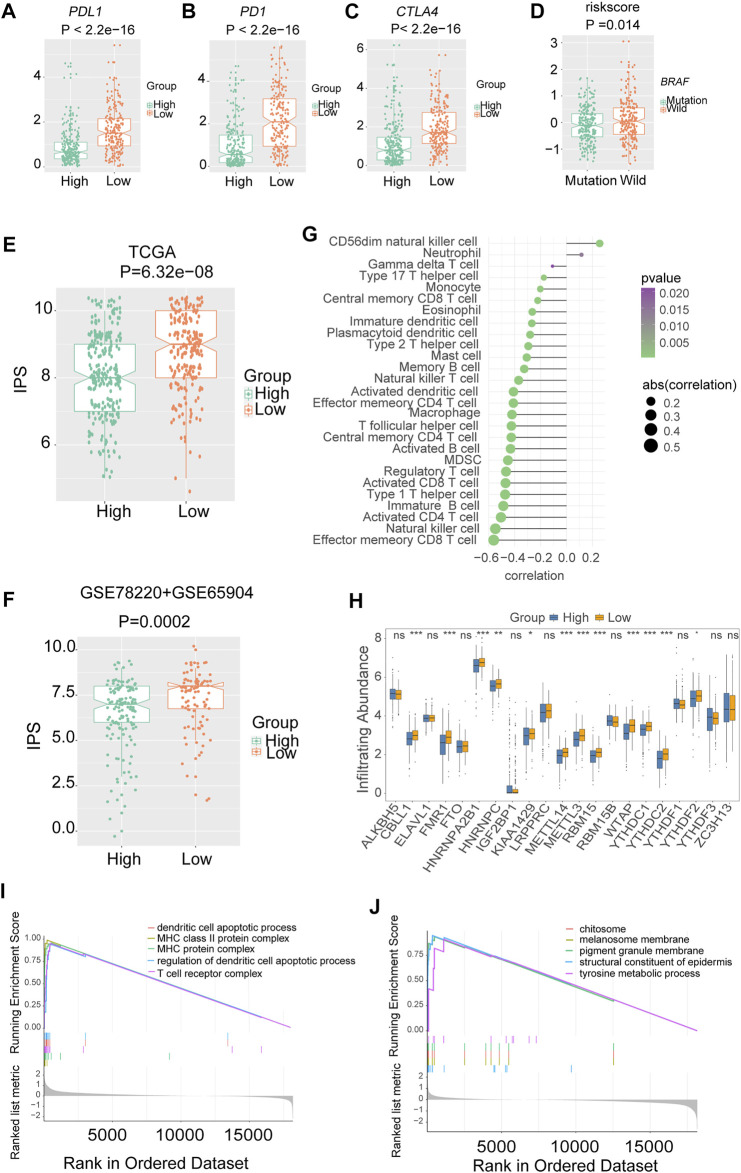
The association between risk signature and immune infiltration in melanoma. The expression of three immune checkpoints in TCGA melanoma with different risk groups **(A)** *PDL1*. **(B)** *PD1*. **(C)** *CTLA4*. **(D)** The level of risk scores in TCGA melanoma with different mutation statuses of *BRAF*. The immunophenoscores (IPS) in TCGA and combined gene expression omnibus (GEO) cohorts, **(E)** TCGA. **(F)** Combined-GEO cohorts. **(G)** The correlation between risk scores and immune infiltration cells in TCGA melanoma. **(H)** Box plots were used to visualize the differential expression of 21 m6A regulators in high- and low-risk groups (*p < 0.05, **p < 0.01, ***p < 0.001). **(I)** Gene ontology (GO) analysis showed that the low-risk group was enriched in pathways associated with immune full activation; **(J)** the high-risk group was prominently enriched in Melanin metabolism pathways.

The low-risk group had higher *PDL1* (Figure 7A), *PD1* (Figure 7B) and *CTLA4* expression (Figure 7C), and immunophenoscore (IPS) (Figures 7E,F) compared with the high-risk group, indicating that the low-risk group may exhibit a better response to immunotherapy. The results were validated in GEO-combined cohorts (Supplementary Figure S9). The correlation between the 28 immune infiltration cells and the risk scores was examined using Spearman correlation analysis (Figure 7G).

Notably, nearly all the writers and readers were significantly upregulated in the low-risk group, indicating the potential correlation of m6A levels and immunotherapy response in melanoma (Figure 7H).

Furthermore, the GO enrichment analysis revealed that the low-risk group was enriched in pathways associated with immune fully activation, including MHC class II protein complex, regulation of dendritic cell apoptotic process, and T cell receptor complex (Figure 7I). In comparison, the high-risk group was prominently related to Melanin metabolism pathways such as melanin biosynthetic process, tyrosine metabolic process, and melanocyte differentiation (Figure 7J). In addition, the risk scores of the *BRAF* wild-type group were higher than that of the mutation group (Figure 7D).

### The Role of Risk Signature in Immune-Checkpoint Blockade Therapy Cohorts

**FIGURE 8:**
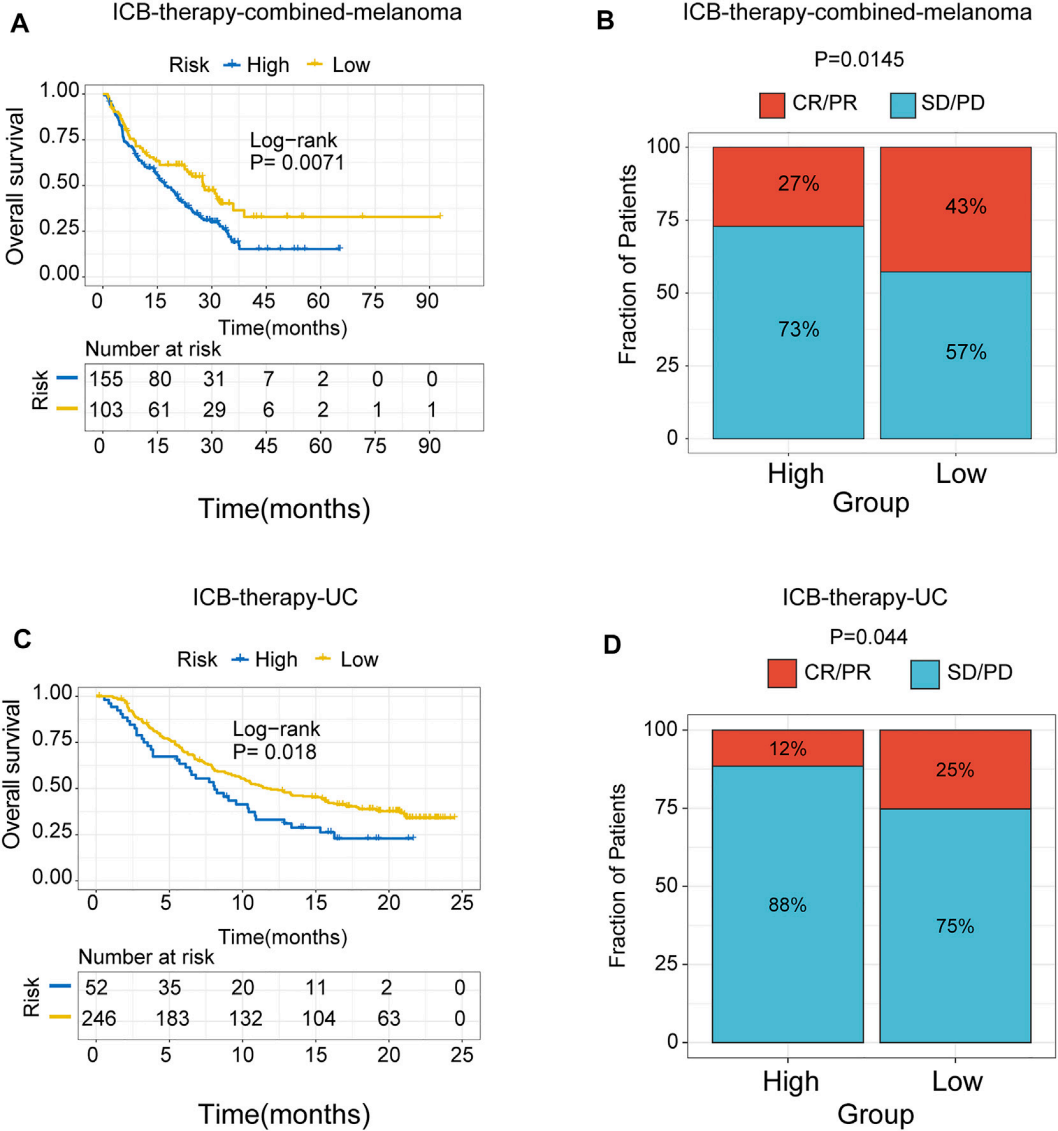
The role of risk signature in ICB therapy cohorts. Kaplan Meier plots of patients treated with immune checkpoint blockade. **(A)** ICB-therapy-combined melanoma. **(B)** ICB- therapy-UC. The degree of immune checkpoint blockade therapy response in high- and low-risk groups. **(C)** ICB-therapy-combined melanoma. **(D)** ICB-therapy-UC. CR, complete response; PR, partial response; SD, stable disease; PD, progressive disease.

To further test the predictive efficiency of m6A-related signature in immunotherapy cohorts, we analyzed the proportion of patients with response to immune checkpoint blockade therapy in low- and high-risk groups. The immunotherapy cohorts included a combined melanoma cohort (ICB-therapy-combined melanoma) and urothelial cancer patients treated with anti-*PDL1* (ICB-therapy-UC). As regards ICB-therapy-combined melanoma, the patients in the low-risk group had a significantly improved survival rate (log-rank test, *p* < 0.05, Figure 8A) and a higher response rate to ICB therapy (Fisher’s exact test, *p* < 0.05, Figure 8B) compared with the high-risk group. Consistent with ICB-therapy-combined melanoma, the patients in the low-risk group exhibited a significantly prolonged survival rate (log-rank test, *p* < 0.05, Figure 8C) and a higher response rate to immune-checkpoint blockade therapy (Fisher’s exact test, *p* < 0.05, Figure 8D) compared with the high-risk group in ICB-therapy-UC.

## Discussion

Although various immunotherapies have revolutionized melanoma treatment, only a few patients can respond well to immunotherapy (Rodriguez-Cerdeira et al., 2017). Recent studies have revealed the multiple functions of m6A methylation in the immune response; however, these studies focused on single m6A regulators. The immune infiltration comprehensively mediated by multiple m6A regulators has never been investigated in melanoma. Therefore, it is necessary to comprehensively evaluate the m6A regulators and immune responses to approach individual treatment.

In the current study, we uncovered a significant correlation between the m6A regulators and immune infiltration in melanoma for the first time. In addition, we identified an m6A-related risk model composed of 12 genes for survival prediction. The methyltransferase *METTL3* controls the function of dendritic cells. The depletion of *METTL3* leads to an elevation in functional maturation damage and phenotypic DC (Wang et al., 2019a). *FTO* plays a crucial role in therapeutic resistance to anti-*PD1* immunotherapy in melanoma. Therefore, one promising strategy is to combine *FTO* inhibition with anti-*PD1* blockade for reducing the resistance to immunotherapy in melanoma (Yang and Wei, 2019b). *METTL3* increased cytokine production *via* a *TLR4*/*NF-kB* signal-induced mechanism indicated that it’s promising to combine m6A and the blockade of immune checkpoints for immunotherapy (Wang et al., 2019b). These studies showed that m6A regulators were key enzymes in shaping different immune infiltration patterns. We stratified the patients into two m6A clusters based on the expression pattern of m6A regulators by consensus clustering analysis. The differences of multiple immune functioning ratings between the two clusters were compared. Considering the strong need to assess the prognosis of patients and effective personalized immunotherapy strategies, we constructed an m6A-related signature with independent prognosis and potential ability to provide immunotherapy strategies. We confirmed the low-risk group had higher *PDL1*, *PD1*, and *CTLA4* expression and immunophenoscore (IPS) than the high-risk group, indicating that the low-risk group may exhibit a better response to immunotherapy. The results were validated in GEO-combined cohorts. The patients in the low-risk group treated with immune checkpoints blockade showed a higher response rate to ICB therapy. Notably, there was trend towards better survival outcome in female patients relative to males in the high- and low-risk groups, respectively (data not shown). These gender differences in outcome were probably related to the inactivation of X chromosome, tumor location, single nucleotide polymorphisms and hormone levels (Machado et al., 2014; Hernando et al., 2016; Klein and Flanagan, 2016; Balaton et al., 2018; Bellenghi et al., 2020; Liu et al., 2021). A prior study showed that m6A methyltransferase proteins RBM15 and RBM15B promote X-inactive specific transcript (XIST)-mediated transcriptional repression (Patil et al., 2016). These interesting observations suggested that m6A RNA methylation is involved in sex-related differences regulation.

There is often no unique “right” answer in the unsupervised clustering method (Arora et al., 2020). Interestingly, K = 2 or 4 were both stable clustering methods according to the consensus matrix and the CDF curve in our study. However, we aim to explore the association between immune infiltration and m6A clusters. Accordingly, we chose the K = 2 for analysis because dichotomous classification maybe a reasonable method to reflect the immune phenotype of “hot” and “cold” tumors (Gajewski et al., 2017; Maleki Vareki, 2018). The binary classification may lead to a better understanding of the property of immune cells infiltration status of the m6A clusters (Supplementary Figure S2). Further, the Calinski-Harabasz (CH) index suggested that K = 2 was the optimal number of groups. In addition, we performed external validation using a combined melanoma cohort to validate the unsupervised clustering model. The result is consistent with the model based on TCGA, and K = 2 was the optimal value. This classification was also verified by further immune phenotype analyses (Supplementary Figure S3). These suggested that K = 2 is the more appropriate method for the identification of m6A clusters in melanoma.

A previous study showed that the hot tumors are enriched with immune infiltration and more likely to benefit from immunotherapy (Jiang et al., 2018). *TGF*β Blocking and anti-*PDL1* antibodies promote T cells to penetrate into the tumor center by decreasing the *TGF*β signal in stromal cells and stimulating anti-tumor immunity and tumor regression (Mariathasan and Turley, 2018a). This is consistent with our result that the low-risk group is enriched in immune activation pathways. Several lines of the study revealed the association between the improved immunotherapy, favorable prognosis, and abundant m6A modification level. Yang et al. showed that knockdown of *FTO* (an eraser) sensitizes melanoma to anti-*PD1* treatment in mice, depending on adaptive immunity (Yang et al., 2019c). Cui et al. demonstrated that overexpression of *METTL3* (a writer) or inhibition of the RNA demethylase *FTO* suppresses Glioblastoma Stem Cells growth and self-renewal (Cui et al., 2017). Our findings also showed the high expression of writers and readers in the low-risk group. In summary, this may have contributed to the high response rate and better prognosis in the low-risk group.

Other m6A-related prognostic signatures also achieved good performance in predicting the prognosis of multiple malignant tumors, including hepatocellular carcinoma, rectum adenocarcinoma, gastric cancer (Guan et al., 2020; Huang et al., 2020; Shen et al., 2020). Compared with other clinical factors (gender, age, TNM stage), the risk signature showed a beneficial supplement to prognostic factors for melanoma. And the m6A-related signature can not only predict melanoma patients’ survival outcomes and predict the response to immunotherapy. These results indicated that m6A-related signature might be a promising predictor for individual treatment.

Previous studies indicated that *BRAF* targeted therapy was associated with improved immune infiltration, such as increased antigen expression, an CD8^+^ T-cell infiltration, class I major histocompatibility complex (MHC) expression (Boni et al., 2010; Frederick et al., 2013; Bradley et al., 2015; Sabbatino et al., 2016). In our result, patients with *BRAF* ^*wild*^ status showed a higher risk score, significantly poor overall survival, progress-free survival, and a trend toward decreased immune score (Supplementary Figure S10). All of the above findings suggested that *BRAF* ^*mut*^ patients with *BRAF* targeted therapy may benefit from immunotherapy due to increased immune infiltration (Naderi-Azad and Sullivan, 2020).

There were several limitations in our study. First, clinical information in our study was relatively incomplete. Furthermore, the data was collected from different platforms, so there is heterogeneity between different data sets and the study population.

In conclusion, our study identified a risk signature that was an independent indicator of prognosis in melanoma. For the first time, we have also found the nonnegligible role of the expression pattern of m6A regulators in shaping immune infiltration in melanoma. However, further experiments are warranted to verify the applicability of the risk signature.

## Data Availability

Publicly available datasets were analyzed in this study. This data can be found here: https://portal.gdc.cancer.gov/, https://www.ncbi.nlm.nih.gov/geo/. The request for the R code used in all analyses should be directed to the corresponding author.

## Abbreviations

AIC, akaike information criterion; CNV, copy number alteration; GEO, gene expression omnibus, GSVA, gene set variation analysis; NRI, net reclassification improvement; ICB, immune-checkpoint blockade; IDI, integrated discrimination improvement; IPS, immunophenoscore; m6A, N6-methyladenosine.
