# Supplementary material for: Identification of an m6A Regulators-Mediated Prognosis Signature For Survival Prediction and Its Relevance to Immune Infiltration in Melanoma
Source: Front Cell Dev Biol. 2021 Nov 25;9:718912. doi: 10.3389/fcell.2021.718912 (PMC8656227; doi:10.3389/fcell.2021.718912)
Supplement: Supplementary file 2 [file DataSheet1.pdf]

Supplemental Information

Figure S1. GO enrichment analysis revealed the potential signaling pathways controlling the mutation patterns of *IGF2BP1*, *KIAA1429*, *YTHDF1***.**

Figure S2. Consensus clustering of 21 m6A regulators identified two m6A clusters of melanoma in TCGA.

Figure S3. Consensus clustering of 21 m6A regulators identified two m6A clusters of melanoma in validation cohort.

Figure S4. The immune infiltration was quantified by estimate and ssGESA algorithms in validation cohort.

Figure S5. Functional annotation for m6A phenotype-related genes using GO enrichment analysis. The color depth of the barplots represented the P.adjust.

Table S1. The association between m6A clusters and clinical indexes in TCGA melanoma cohort(n=457).

Table S2. Summary of differentially expressed genes between the two m6A clusters (P < 0.05 and | logFC | >0.5)

Table S3. The results of univariate Cox analyses.

Table S4. The coefficients of the twelve genes in the m6A-related signature.

Figure S6. Receiver operating characteristic (ROC) curves and area under the curve (AUC) values for risk signatures in the 1st and 3rd year.

Figure S7. Forest plot representation of the multivariate Cox regression analysis of risk signature with age, gender and tumor stage were taken into account.

Figure S8. The comparation between old model (AJCC stage only, without riskscore) and combined model (combined stage and riskscore).

Figure S9. Expression of three immune checkpoints genes in three GEO-combined validation set.

Figure S10 Kaplan Meier plots and Immunescore of TCGA melanoma patients with different mutation statuses of *BRAF*.

Figure S1.

a

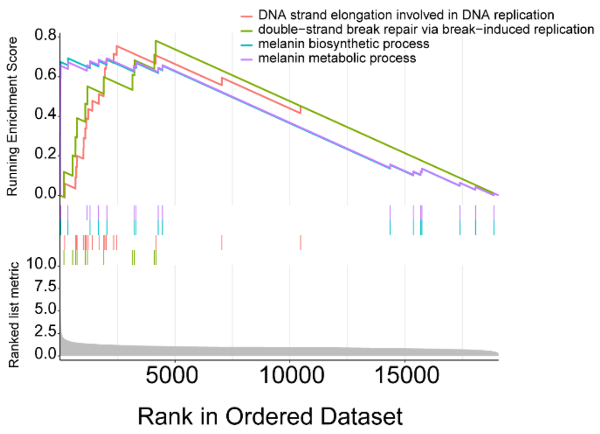

b

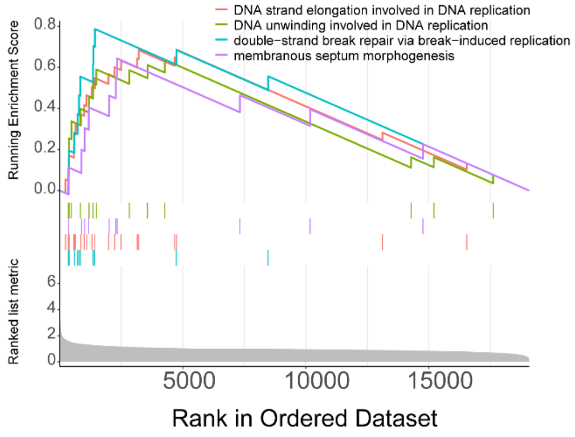

c

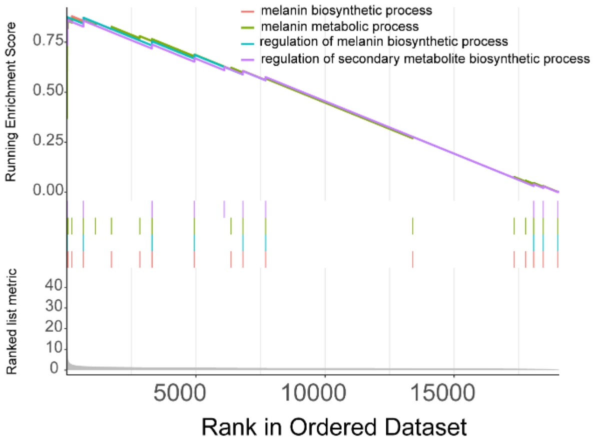

Figure S1. GO enrichment analysis revealed the potential signaling pathways controlling the mutation patterns of *IGF2BP1*, *KIAA1429*, *YTHDF1*. **(a)** *IGF2BP1*. **(b)** *KIAA1429*. **(c)** *YTHDF1*

Figure S2.

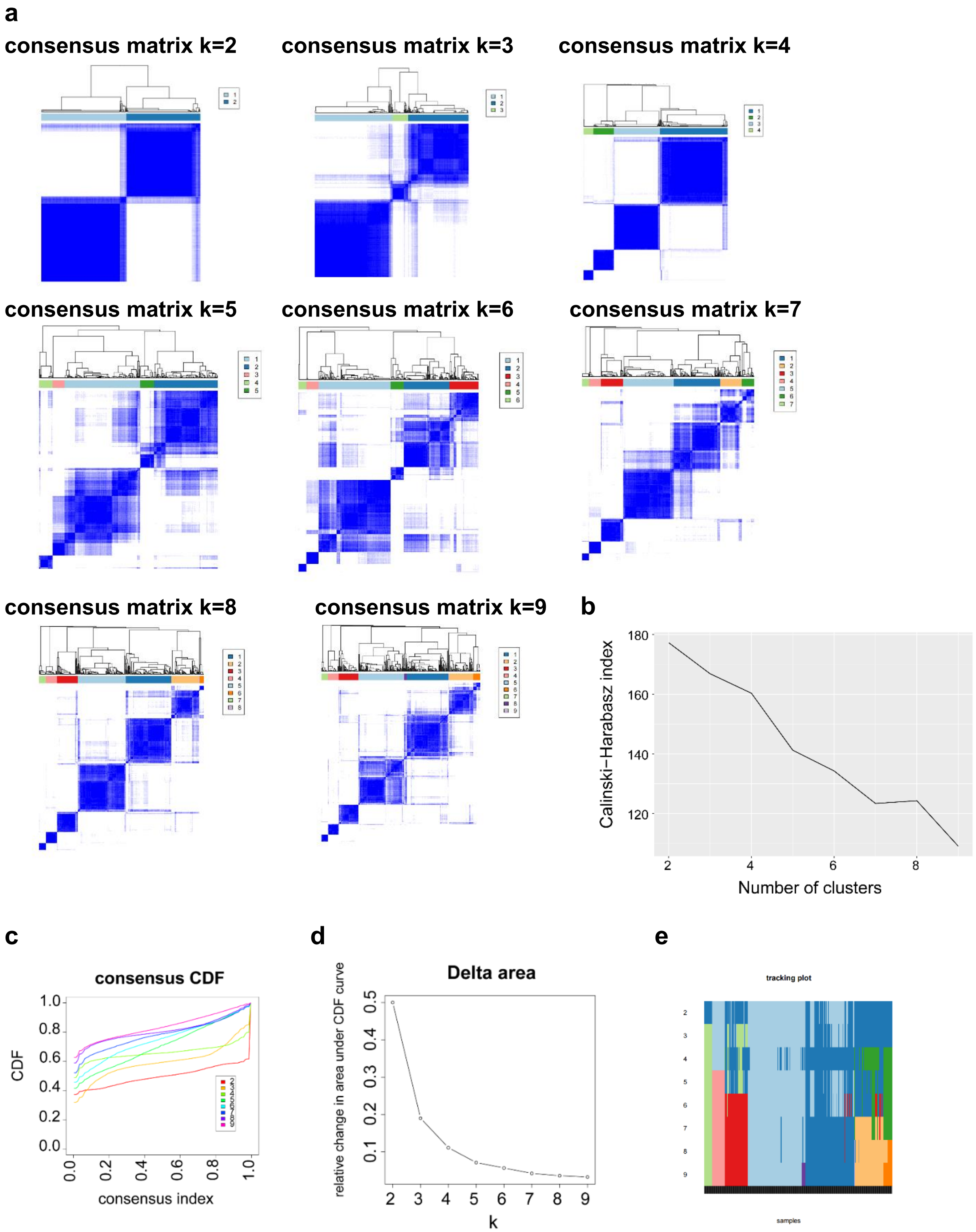

Figure S2. Consensus clustering of 21 m6A regulators identified two clusters of melanoma in TCGA. (a) Consensus clustering matrix for k = 2 to k = 9. **(b)** The Calinski-Harabasz index when k=2 to 9. **(c)** Consensus clustering cumulative distribution function (CDF) when K=2 to 9. **(d)** The relative change of area under the CDF curve when K=2 to 9. **(e)** The tracking plot for k = 2 to k = 9.

Figure S3

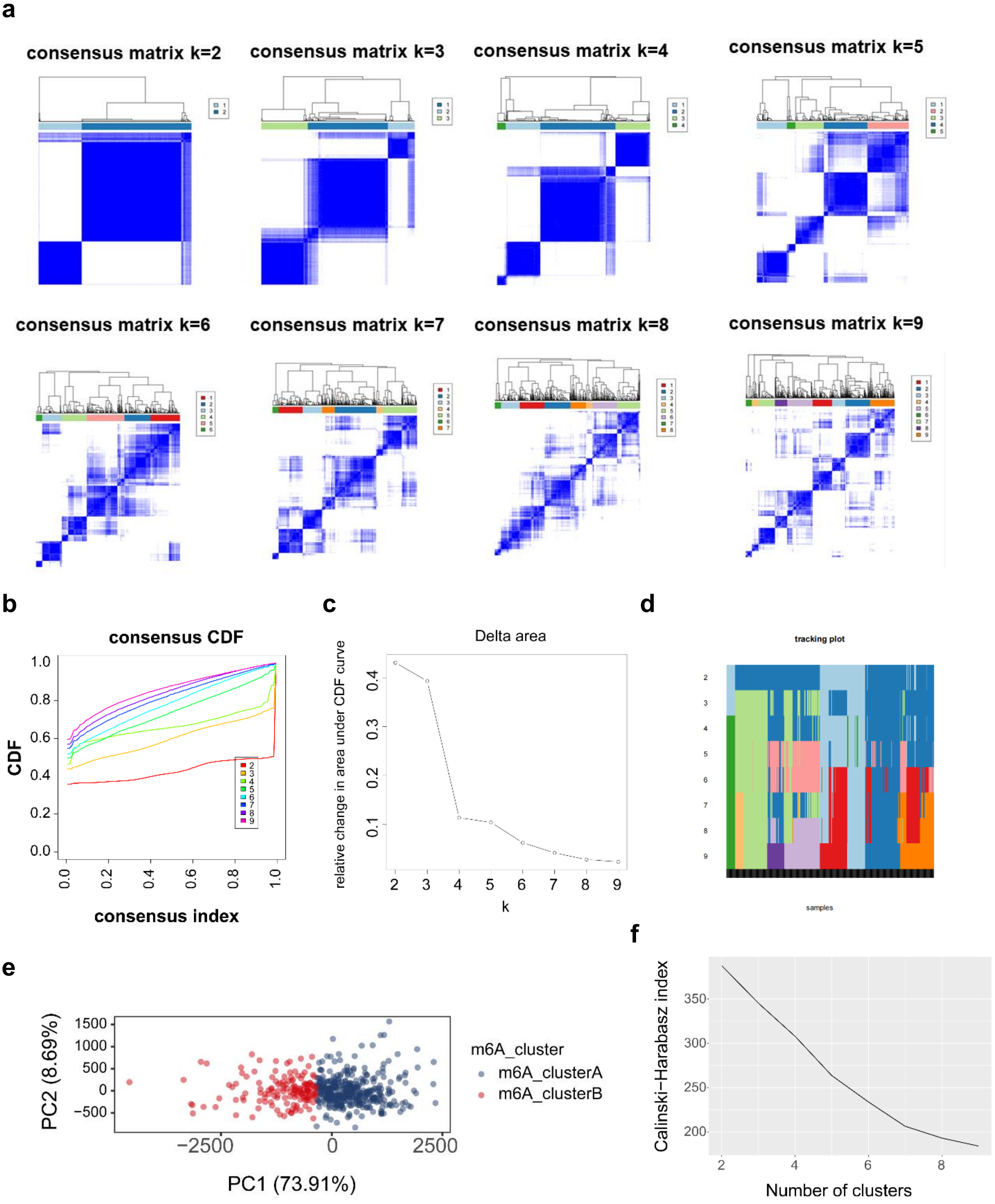

Figure S3. Consensus clustering of 21 m6A regulators identified two m6A clusters in validation cohort. (a) The consensus matrix when K=2 to 9. **(b)** Consensus clustering cumulative distribution function (CDF) when K=2 to 9. **(c)** The relative change of area under the CDF curve when K=2 to 9. **(d)** The tracking plot for k = 2 to k = 9. **(e)** Principal component analysis indicated that there was a significant difference in transcriptome between the two m6A clusters. **(f)** The Calinski-Harabasz index when k=2 to 9.

Figure S4

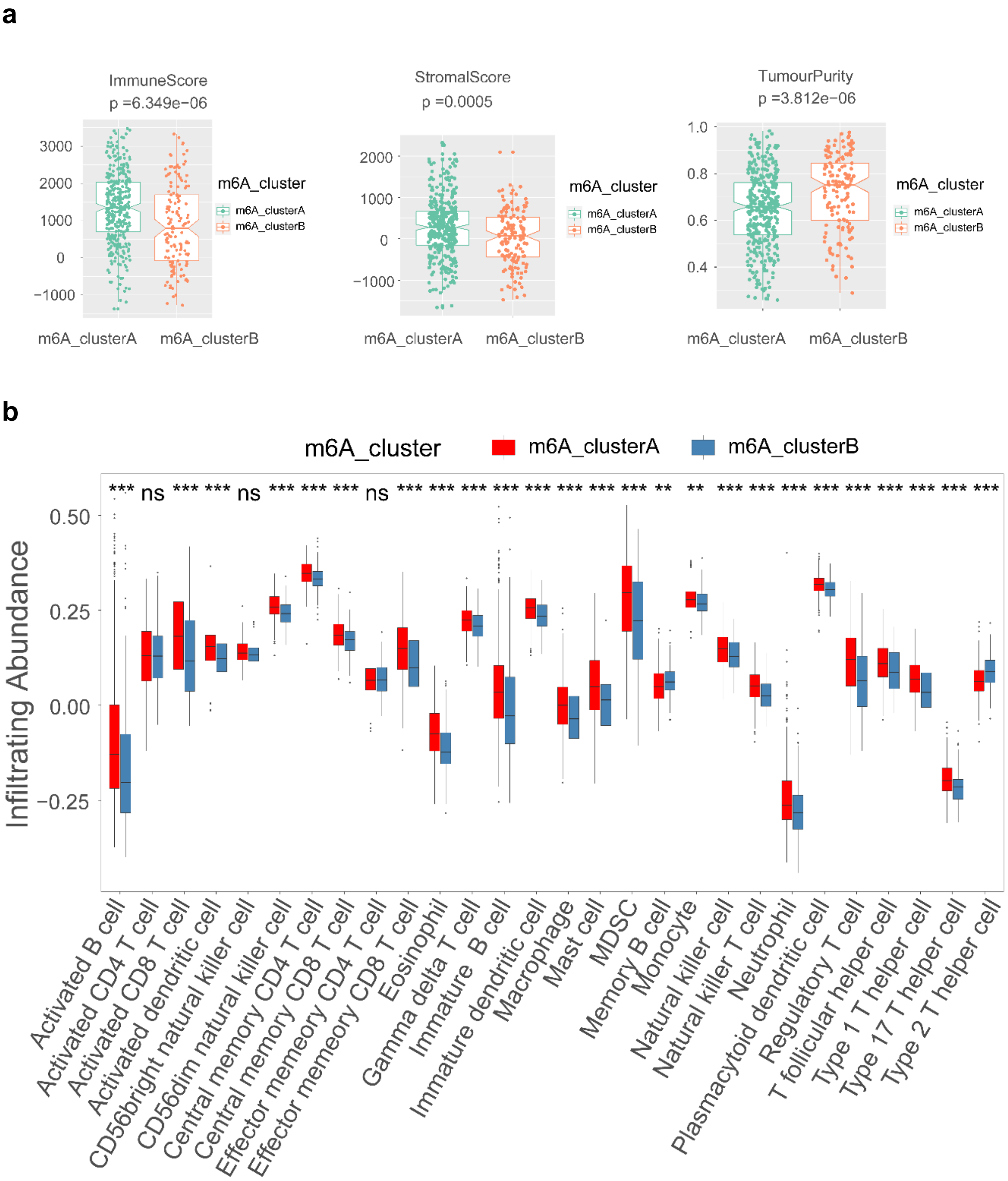

Figure S4. The immune infiltration was quantified by estimate and ssGESA algorithms in validation cohort. **(a)** Immune scores in melanoma samples were compared between the two m6A clusters (left). Stromal scores in melanoma samples were compared between the two m6A clusters (middle). Tumor purity in melanoma samples was compared between the two m6A clusters (right). **(b)** The infiltration of 28 immune cells type identified by single-sample gene set enrichment analysis (ssGSEA) between the two m6A clusters.

Table S1. The association between m6A clusters and clinical indexes in TCGA melanoma cohort(n=457)

TCGA	m6A-clusterA	m6A-clusterB	P
Total	245	212	
**Age**			0.564
≤60	131	120	
>60	114	92	
**Gender**			0.1581
Female	100	72	
Male	145	140	
**TNM Stage**			0.565
Stage I-II	114	99	
Stage III-IV	107	86	
NA	24	27	
FMR1[a]			**2.481e-10**[b]
Low	157	72	
High	88	140	
METTL3 [a]			**8.222e-10**[b]
Low	156	73	
High	89	139	
YTHDC2 [a]			**2.036e-11**[b]
Low	159	70	
High	86	142	

[a] m6A regulators with survival significance

[b] Indicate statistically significant (chi-square test, P<0.05)

Figure S5.

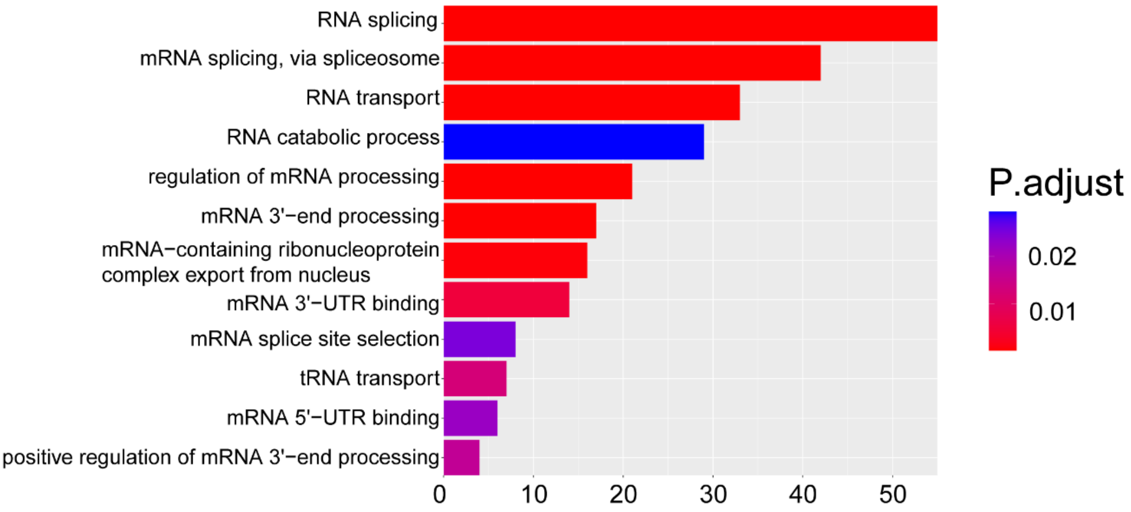

Figure S5. Functional annotation for m6A phenotype-related genes using GO enrichment analysis. The color depth of the barplots represented the P.adjust.

Table S2. Summary of differentially expressed genes between the two m6A clusters (P < 0.05 and | logFC | >0.5). The results are shown in excel.

Table S3. The results of univariate Cox analyses. The results are shown in excel.

Table S4. The coefficients of the twelve genes in the m6A-related signature.

Gene	Coefficients
IL6ST	0.201163
MBNL1	0.277527
NXT2	-0.12676
EIF2A	-0.26691
CSGALNACT1	-0.24339
C11orf58	-0.20566
CD14	0.365687
SPI1	-0.39669
NCCRP1	0.204668
BOK	0.203181
CD74	-0.43106
PAEP	0.213546

Figure S6.

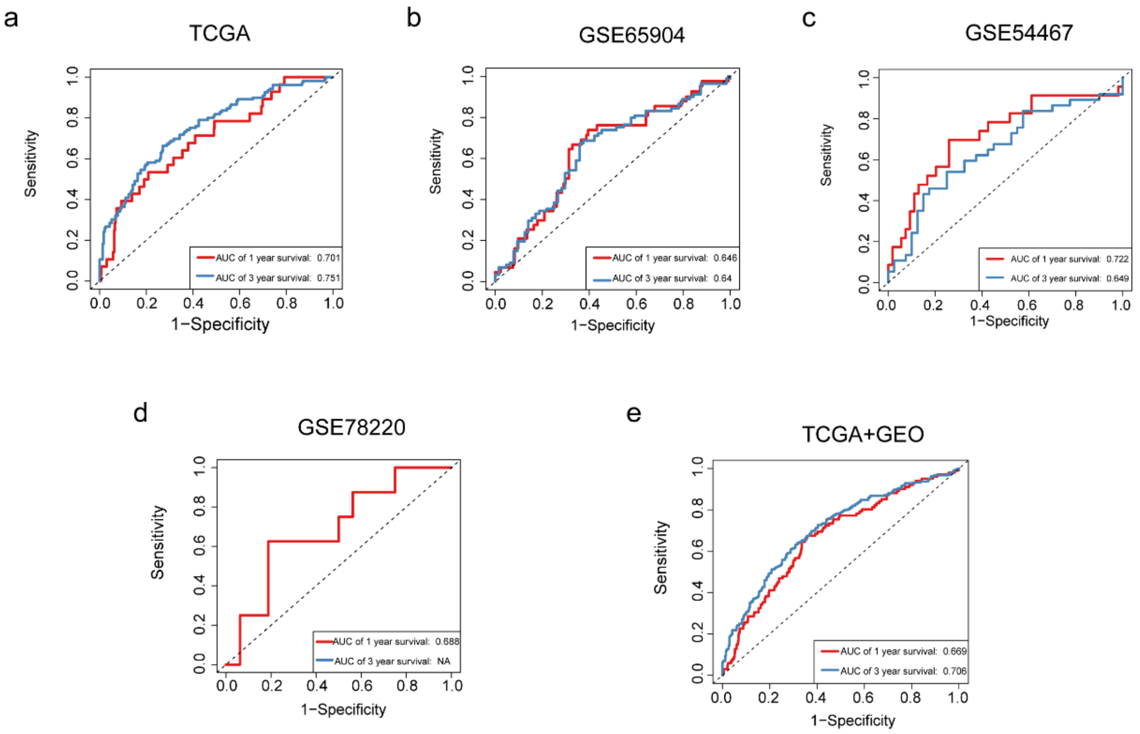

Figure S6. Receiver operating characteristic (ROC) curves and area under the curve (AUC) values for risk signatures in the 1st and 3rd year. (a) TCGA. **(b)** GSE65904. **(c)** GSE54467. **(d)** GSE78220. **(e)** TCGA+GEO.

Figure S7

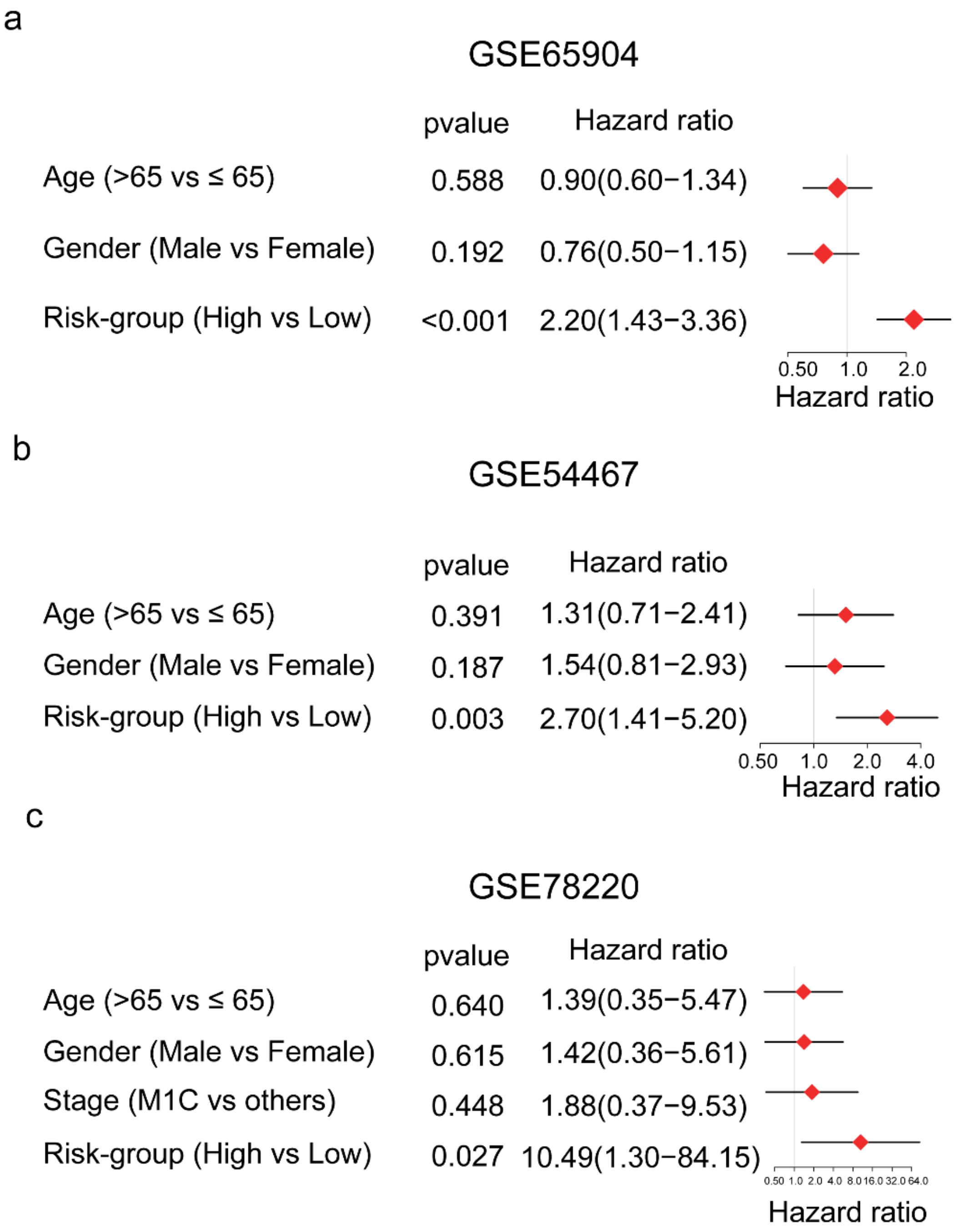

Figure S7. Forest plot representation of the multivariate Cox regression analysis of risk signature with age, gender and tumor stage were taken into account. **(a)** GSE65904, **(b)** GSE54467, **(c)** GSE78220.

Figure S8

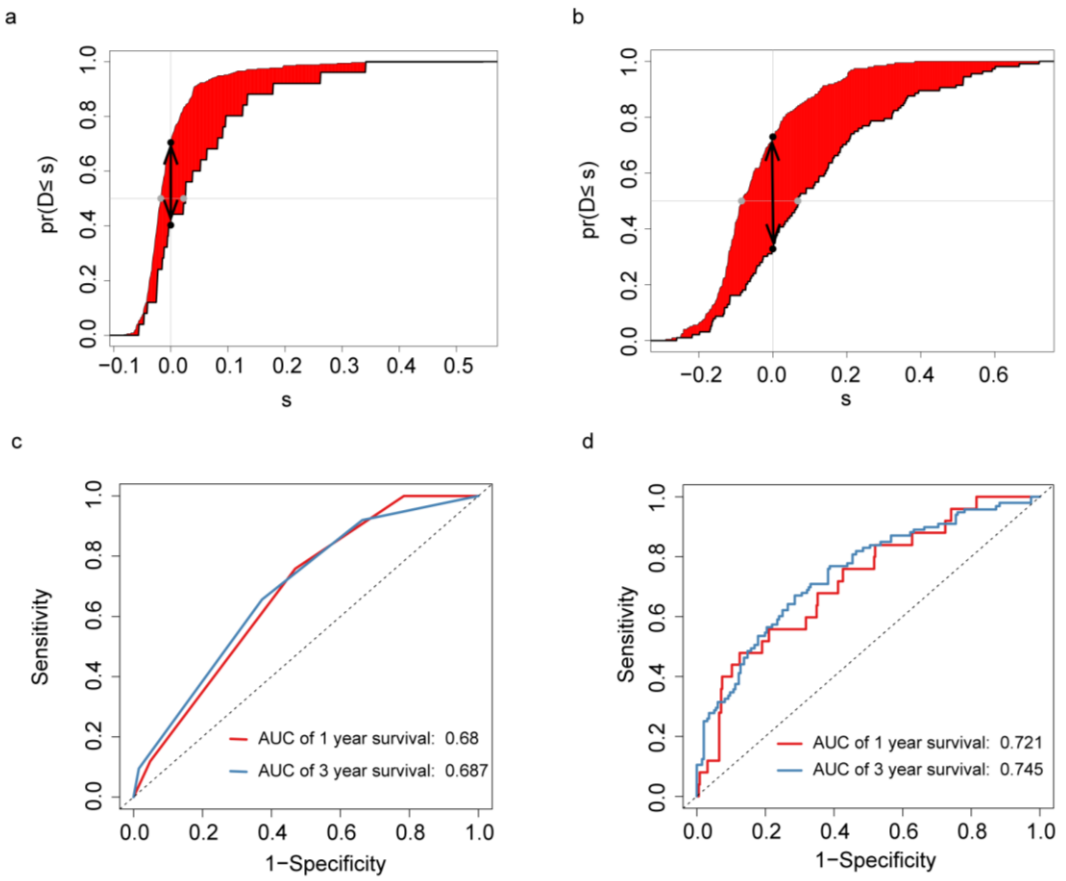

Figure S8. The comparation between old model (AJCC stage only, without riskscore) and combined model (combined stage and riskscore). IDI and NRI for **(a)** 1- and **(b)** 3-years in old model and combined model. The area of red showed the IDI. The distance between two black blots showed the NRI. ROC curves and AUC values for **(c)** old model and **(d)** combined model in the 1- and 3- years.

Figure S9.

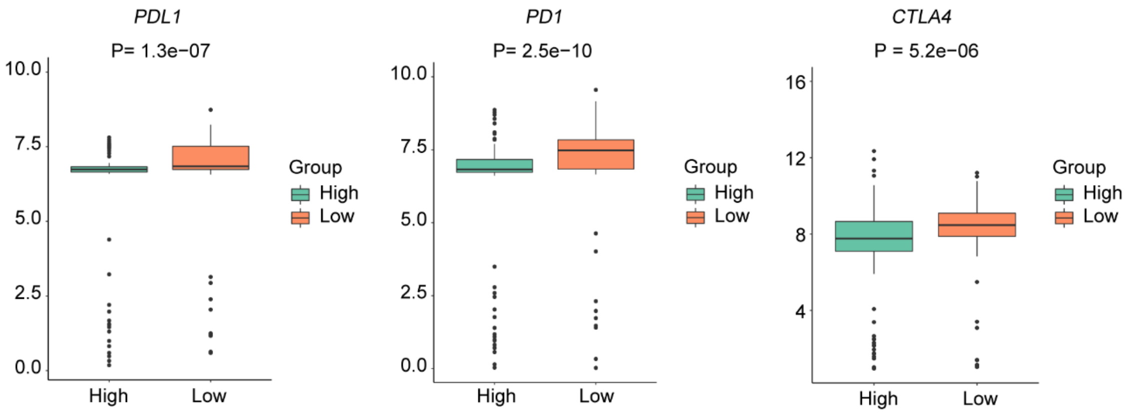

Figure S9. Expression of three immune checkpoints genes in three GEO-combined validation set. *PDL1* (left), *PD1* (middle), *CTLA4* (right).

Figure S10.

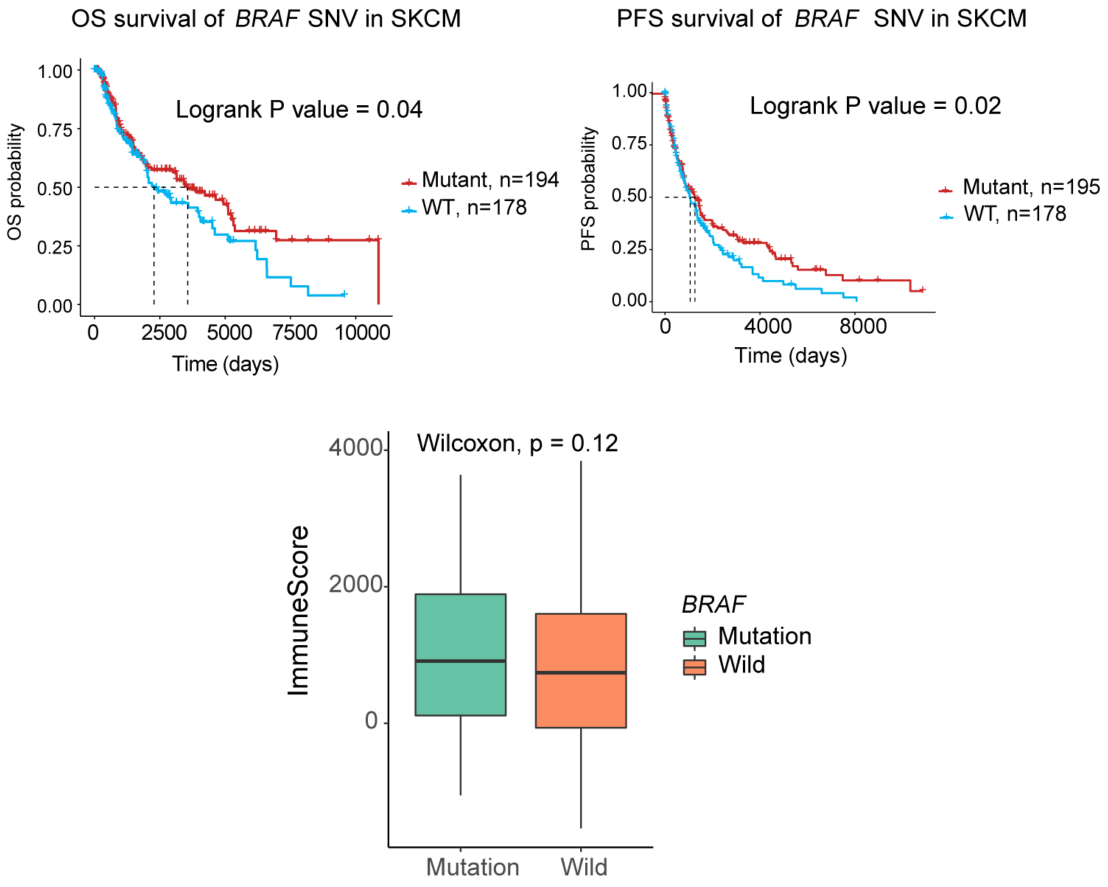

Figure S10. Kaplan Meier plots and Immunescore of TCGA melanoma patients with different mutation statuses of *BRAF*. (a) OS survival. **(b)** PFS survival. **(c)** Immunescore in melanoma samples were compared between the *BRAF*^{mut} and *BRAF*^{wild} subgroup.
